# An Unusual Case of Cardiac Tamponade From a Foreign Body

**DOI:** 10.1016/j.jaccas.2025.105933

**Published:** 2025-12-17

**Authors:** Allen Zheng, Aditya Sengupta, Ahmed El-Eshmawi

**Affiliations:** Department of Cardiovascular Surgery, Icahn School of Medicine at Mount Sinai, New York, New York, USA

**Keywords:** pericardial effusion, tamponade

**Figure:**
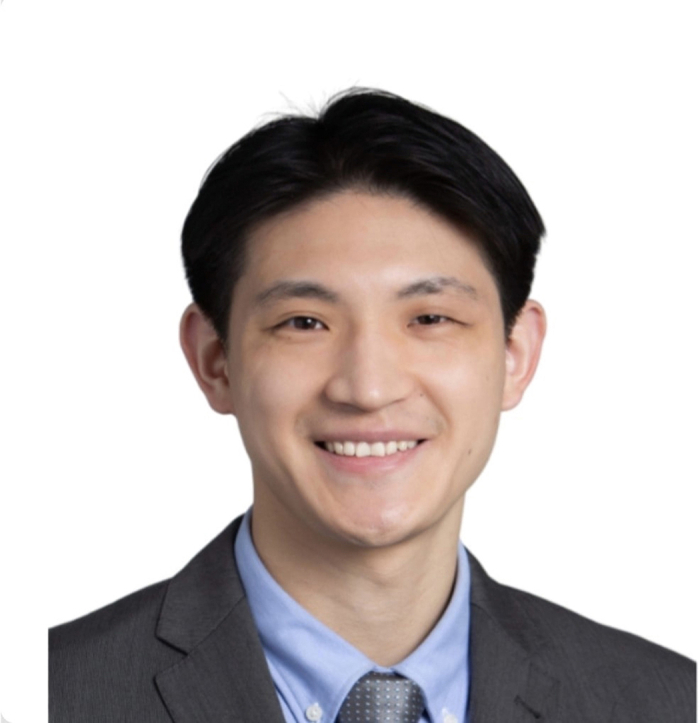

The case presented by Ng et al in this issue of *JACC: Case* *Reports*^1^ describes a rare cause of hemorrhagic pericardial tamponade—a retained hypodermic needle that was ultimately found to be lodged in the left lobe of the liver abutting the right ventricle. While the site of entry was unknown, the authors suggest that the needle fragment had migrated through soft tissue, ultimately traversing through the liver and piercing the pericardium. While there have been reports of needles migrating through soft tissue and causing myocardial injury,^2^ the present case is perplexing as the needle was located 60 mm from the skin surface, and there was no evidence of liver dysfunction. Alternatively, the fragment could have migrated through the vasculature to end up in the right ventricle, whereupon it traversed the free wall and pericardium to embed itself in the liver. Regardless of directionality, after the needle entered the pericardial space, a hemorrhagic effusion developed that led to tamponade and impending cardiovascular collapse.

This case highlights several key concepts in acute cardiac care, including the importance of a broad differential diagnosis, swift recognition of tamponade physiology, and effective multidisciplinary collaboration between medical and surgical teams for complex cases. While the initial presentation of chest pain with ST-segment elevations after cocaine usage is naturally suspicious for coronary vasospasm or an acute coronary syndrome, global ST changes with a relatively clean coronary angiogram and pulsus paradoxus quickly prompted workup for pericardial tamponade.

Given that the needle was embedded in the liver and that pericardiocentesis had managed to resolve the hemodynamic instability with no evidence of reaccumulation, an abdominal approach to extract the needle with cardiothoracic surgery on standby was reasonable. In this case, it was unclear if there was puncture of the right ventricle. In general, any concern for penetrating cardiac injury warrants surgical exploration of the heart. While the low-pressure system of the right ventricle can sometimes facilitate closure of small punctures without intervention, this should not be assumed. Patients who do not undergo surgical exploration should be monitored closely with serial echocardiography.

Retained intravascular or intracardiac foreign bodies are rare but well-recognized in the surgical literature. Hypodermic needle fragmentation is a well-known complication associated with intravenous drug use, especially in the context of needle reuse. Needle retention can lead to infection or, rarely, embolization to the cardiopulmonary system. There have been several case reports of the latter that describe needle fragments embedding themselves in the right ventricle^3^^,^^4^ or pulmonary vasculature.^5^ Early removal of these structures is generally indicated in order to prevent infection or perforation, which can be catastrophic.

This report adds to the sparse but important literature on retained foreign bodies in proximity to the heart and their associated complications. The medical team should be commended for their timely diagnosis and effective management.

## Funding Support and Author Disclosures

The authors have reported that they have no relationships relevant to the contents of this paper to disclose.
